# Editorial: Hedgehog signaling pathway in development and cancer

**DOI:** 10.3389/fcell.2022.1010230

**Published:** 2022-08-31

**Authors:** Steven Y. Cheng, Matthias Lauth, Aimin Liu

**Affiliations:** ^1^ Department of Medical Genetics, School of basic medical sciences, Nanjing Medical University, Nanjing, Jiangsu, China; ^2^ Philipps University Marburg, Department of Gastroenterology, Endocrinology and Metabolism, Center for Tumor- and Immune Biology, Marburg, Germany; ^3^ Department of Biology, Eberly College of Science, The Pennsylvania State University, University Park, PA, United States

**Keywords:** hedgehog, smoothened, GLI = glioblastoma transcription factor, development, Cancer

The hedgehog (HH) family of secreted proteins, and the signaling pathway they trigger, play crucial roles in cell proliferation, differentiation and migration, not only in development, but also in many types of cancers. It is thus not surprising that it has been the focus of intense investigation by biochemists, geneticists and clinical doctors. Four decades of studies have revealed many levels of regulation, from the production, modification, release and degradation of the ligands, to the roles of various receptors, coreceptors, downstream kinases, phosphatases, and effector transcription factors. Strikingly, HH signaling in vertebrates has been associated with the primary cilium, a unique cell surface organelle whose critical roles in development and diseases were only appreciated in the past two decades. The importance of this pathway is further exemplified by the FDA approval of HH/SMOOTHENED (SMO)-blockers as a stand-alone anti-cancer therapy. To date, novel regulators and effectors of HH signaling continue to be identified, adding to the complexity of the molecular interaction network.

The seven articles in this Research Topic reflect on the recent advances and provide perspectives in the molecular mechanisms of HH pathway regulation, the crucial roles of HH signaling in development and pathology, as well as new strategies for treating HH signaling related diseases including cancers.

Given its significance for correct embryonic development and its involvement in the patterning of various tissues and organs, HH signaling is subject to tight regulation. One such layer of regulation is imposed by distinct lipid compositions specifying selected membrane regions as found in the primary cilium of vertebrates. Nguyen et al. provide an overview on ciliary lipid compositions and their impact on HH signaling throughout evolution (Nguyen et al.). In addition to ciliary membrane-specific phosphoinositides, this signaling pathway is stunningly dependent on many other lipids and sterols, functionally affecting HH ligand transport as well as intracellular signal transduction—a truly unique feature of the HH pathway.

One example for the intricate relationship between lipids and signaling is the SMO protein, which can be covalently and non-covalently associated with sterols. However, SMO is also subject to ubiquitination and SUMOylation, both of which constitute evolutionarily conserved control mechanisms of HH signaling in *Drosophila* and mammals. Jia and Jiang outline the regulation of SMO trafficking by these post-translational modifications and hereby compare mechanistic concepts which arose in flies and humans (Jia and Jiang).

The Glioma associated oncoprotein (GLI) family of transcription factors are the effectors of the HH pathway, converting the HH signal into transcriptional responses leading to cellular outputs. Zhou and Jiang provide a review on phosphorylation as a major mechanism of GLI regulation (Zhou and Jiang). They introduce our current understanding of cAMP-dependent protein kinase (PKA), Casein kinase 1 (CK1) and Glycogen synthase kinase 3 (GSK3)-mediated phosphorylation in the processing and activation of GLI proteins in vertebrates and invertebrates. They also discuss the latest research findings, some of which are still being debated, e.g., on the roles and regulations of ciliary cAMP in HH signaling. They also discuss the discovery of new GLI regulators, such as the Fused family of kinases, Polo-like kinase 1 (PLK1) and CK2, which catalyze the phosphorylation of GLI proteins, and contribute to their functional regulation.

Micro RNA (miRNA) regulation of HH signaling has been an intense ResearchTopic over the past decade; however, the preferred genetic loss of function approach often yields dismally little useful information, possibly due to functional redundancy and contextual dependence of this class of regulators as a whole or each miRNA in particular. Here, He et al. report a study carried out in *Drosophila*, in which they generated a transgenic toolbox of *in vivo* miRNA sensors for the core components of the HH pathway, and performed a genome-wide *in vivo* miRNA overexpression screen in the developing wing imaginal disc (He et al.). Out of 12 miRNAs identified through this screening, they report that miR-10 and miR-958 attenuate HH signaling by directly targeting *fu* and *smo*, respectively. The functional relevance of the newly discovered miRNAs was validated by overexpression experiments that altered HH’s role in cell fate specification during testis development.

HH signaling plays essential roles in the development of many organs, and when abnormally activated, leads to malignancies. Wang et al. review the roles of HH signaling in cerebellar development and cancer (Wang et al.). They review classical genetic loss-of-function studies establishing SHH-mediated interaction between Purkinje cells (PC) and granule cell progenitors (GCP), two major neuronal cell types of the cerebellum. They focus on GLI targets that directly regulate cell proliferation, including Cyclin D (CycD), Cyclin-dependent kinase 4 (Cdk4), MYCN, and their roles in the pathogenesis of medulloblastoma (MB), a pediatric cerebellar tumor. Finally, they discuss the use of inhibitors for SMO, GLI and downstream CDKs as therapeutic strategies for MBs. Some of the inhibitors are already approved or in clinical trials for MB treatment, attesting to the clinical importance of understanding the roles of HH signaling in cerebellar development and MB etiology.

Tesanovic et al. present another example of the importance for well-orchestrated HH signaling in normal developmental processes, and how its dysregulation leads to malignancies such as acute myeloid leukemia (AML), one of the most lethal types of blood-borne cancers (Tesanovic et al.). HH signaling is critical for the persistence of therapy-refractory leukemic stem cell pools and a SMO inhibitor has recently been approved for clinical use in AML, significantly expanding the application repertoire of this class of compounds beyond that of basal cell carcinoma.

Another HH family member, Indian hedgehog (IHH), plays a critical role in chondrogenesis in development. Ma et al. studied the roles of parathyroid hormone (PTH) in fracture healing using a mouse model (Ma et al.). They show that endogenous PTH is required for efficient fracture healing, and a shortened exogenous PTH fragment (PTH 1–34) can rescue the fracture healing defects in *Pth* mutant mice. Importantly, they find that PTH 1–34 promotes cAMP response element binding protein (CREB) phosphorylation and subsequent activation of *Ihh* expression. This work suggests that PTH 1–34 can be an effective treatment for fracture, and points to an important role of IHH signaling as the underlying mechanism.

In summary, the seven articles, authored by experts at the forefront of the HH signaling field, cover a wide range of research areas, from basic mechanisms of HH signal transduction and pathway regulation, to the connection between HH signaling and various diseases. Finally, current and potential treatments of various cancers as well as of bone fracture repair are presented. Hence this Research Topic provides a timely update on the latest progress in the study of HH signaling in development and disease, and points out pressing questions and future directions.

